# Prognostic value of the early lung ultrasound B-line score for postoperative pulmonary insufficiency in patients undergoing thoracic surgery: an observational study

**DOI:** 10.1186/s40001-023-01117-3

**Published:** 2023-05-03

**Authors:** Yipeng He, Xiaoxiao Xu, Chenhao Wang, Zhouquan Wu

**Affiliations:** 1 Department of Anesthesiology, Nanjing Medical University Affiliated, Changzhou No. 2 People’s Hospital, Changzhou, 213003 China; 2 Graduate School of Dalian Medical University, Liaoning, 116044 China

**Keywords:** Lung ultrasound, B-line, Postoperative pulmonary insufficiency, Oxygenation index

## Abstract

**Background:**

Postoperative pulmonary insufficiency (PPI) is an important contributor to morbidity and mortality after thoracic surgery. Lung ultrasound is a reliable tool for assessing respiratory function. We sought to determine the clinical value of the early lung ultrasound B-line score for predicting changes in pulmonary function after thoracic surgery.

**Methods:**

Eighty-nine patients undergoing elective lung surgery were included in this study. The B-line score was determined 30 min after removal of the endotracheal tube, and the PaO_2_/FiO_2_ ratio was recorded 30 min after extubation and on the third postoperative day. Patients were divided into normal (PaO_2_/FiO_2_ ≥ 300) and PPI (PaO_2_/FiO_2_ < 300) groups according to their PaO_2_/FiO_2_ ratios. A multivariate logistic regression model was used to identify independent predictors of postoperative pulmonary insufficiency. Receiver operating characteristic (ROC) analysis was performed for significantly correlated variables.

**Results:**

Eighty-nine patients undergoing elective lung surgery were included in this study. We evaluated 69 patients in the normal group and 20 in the PPI group. Patients conforming to NYHA class 3 at administration were significantly more represented in the PPI group (5.8 and 55%; *p* < 0.001). B-line scores were significantly higher in the PPI group than in the normal group (16; IQR 13–21 vs. 7; IQR 5–10; *p* < 0.001). The B-line score was an independent risk factor (OR = 1.349 95% CI 1.154–1.578; *p* < 0.001), and its best cutoff value for predicting PPI was 12 (sensitivity: 77.5%; specificity: 66.7%).

**Conclusions:**

Lung ultrasound B-line scores 30 min after extubation are effective in predicting early PPI in patients undergoing thoracic surgery.

*Trial registration* This study was registered with the Chinese Clinical Trials Registry (ChiCTR2000040374).

**Supplementary Information:**

The online version contains supplementary material available at 10.1186/s40001-023-01117-3.

## Introduction

Pulmonary complications after thoracic surgery, including atelectasis, pneumonia, pleural effusion, persistent bronchopleural fistula, empyema, pulmonary embolism and pulmonary insufficiency, occur in up to 30% to 50% of patients and can lead to postoperative mortality and morbidity [1, 2]. However, postoperative pulmonary insufficiency (PPI) is still the major cause of morbidity and significantly affects several aspects of morbidity, including the length of hospital stay and unexpected intensive care unit admissions [3–5]. Several risk stratification models for PPI have described patient-specific risk factors, including age, sex, preoperative hypoalbuminemia, anemia and surgical or anesthesiological factors, including the extent of resected lung tissue, surgical time, surgical approach via thoracotomy versus video-assisted thoracoscopy, excessive fluid administration, inappropriate ventilation strategies, and inadequate postoperative analgesia [6–10]. The interaction of the above risk factors can lead to a large amount of fluid entering the third space and even obvious pulmonary interstitial edema in severe patients, which ultimately provides a pathophysiological basis for the occurrence of PPI. To date, there is no clear risk score for making a wise evaluation of PPI after lung surgery. Although the ARISCAT Score is a well-established risk score for postoperative pulmonary complications, it is not specific for lung surgery [1].

As a noninvasive tool providing almost unlimited repetitions, lung ultrasound has become a valuable method for evaluating pleural effusion and pneumothorax in critical care [11–13], and there are complex schemes for diagnosing various causes of respiratory insufficiency [14, 15]. As an important part of lung ultrasound, the B-line score is a new method that is used to quantify the interstitial fluid of the lung. The formation of a B-line on lung ultrasound reflects increased density of local lung tissue and decreased ventilation, thus suggesting the presence of pulmonary complications. When pulmonary edema (e.g., cardiogenic or acute respiratory distress syndrome), pulmonary inflammation (parenchymal or interstitial), and diffuse pulmonary parenchymal lesions (e.g., pulmonary fibrosis) occur, multiple B-lines different from those seen in patients with normal lung function will appear [16]. Notably, the formation of a diffuse B-line is more common in patients with pulmonary edema caused by extravascular pulmonary hydration [17]. As there has been no previous study regarding the use of the B-line score to predict the prognosis of pulmonary surgery, the purpose of this study was to determine whether early B-line values could predict postoperative pulmonary function in patients undergoing such surgery.

## Materials and methods

### Patients and study design

**Fig. 1:**
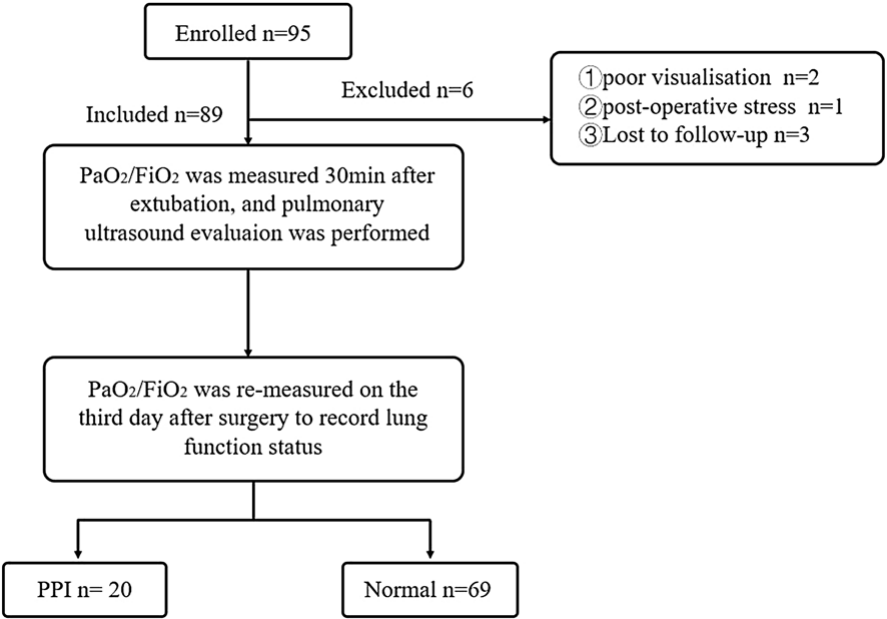
Flowchart demonstrating patient selection

This study was approved by the Ethics Committee of The Affiliated Changzhou No. 2 People’s Hospital of Nanjing Medical University ([2020]KY134-0l) and registered with the Chinese Clinical Trials Registry (ChiCTR2000040374). Written informed consent was obtained from all patients. Patients described as ASA classification 1 to 3 who were undergoing elective lung surgery under general anesthesia at The Affiliated Changzhou No. 2 The People’s Hospital of Nanjing Medical University between December 15, 2020, and March 1, 2021, was considered for inclusion in this prospective cohort study. Patients were excluded from the study if they had pre-existing pulmonary fibrosis, if they were undergoing several surgeries during hospitalization, if they were undergoing cardiac surgery, if they had a thoracic deformity (leading to difficult image acquisition), or if they had obvious postoperative stress factors (e.g., bleeding, severe pain, severe arrhythmia). Eligible study patients were then divided into two groups based on the ratio of partial pressure of arterial oxygen to fraction of inspired oxygen (PaO_2_/FiO_2_) 3 days after surgery [3, 18–20]: the normal group (PaO_2_/FiO_2_ ≥ 300) and the pulmonary insufficiency (PPI) group (PaO_2_/FiO_2_ < 300) [21] (Fig. 1).

### Anesthesia

For induction of anesthesia, patients were administered sufentanil 1 μg/kg, propofol 2 mg/kg, and rocuronium 0.9 mg/kg. Midazolam 0.1 mg/kg was also administered. For maintenance of anesthesia, patients were given 1% sevoflurane by continuous inhalation, remifentanil 0.2 ug/kg/min, propofol 5 mg/kg/h, and cisatracurium 1 mg/kg/h. The dose rate of all medications was dynamically adjusted to maintain the bispectral index at 40 to 60. A double-lumen tube was used to achieve single-lung ventilation. A pressure controlled-volume guaranteed ventilation mode was used, with tidal volume set at 4 to 6 mL/kg and airway peak pressure < 30 mbar. The respiratory rate was adjusted to maintain an end-tidal carbon dioxide level between 35 and 45 mmHg. Epidural analgesia was administered to all patients.

### Lung ultrasound B-line score

**Fig. 2:**
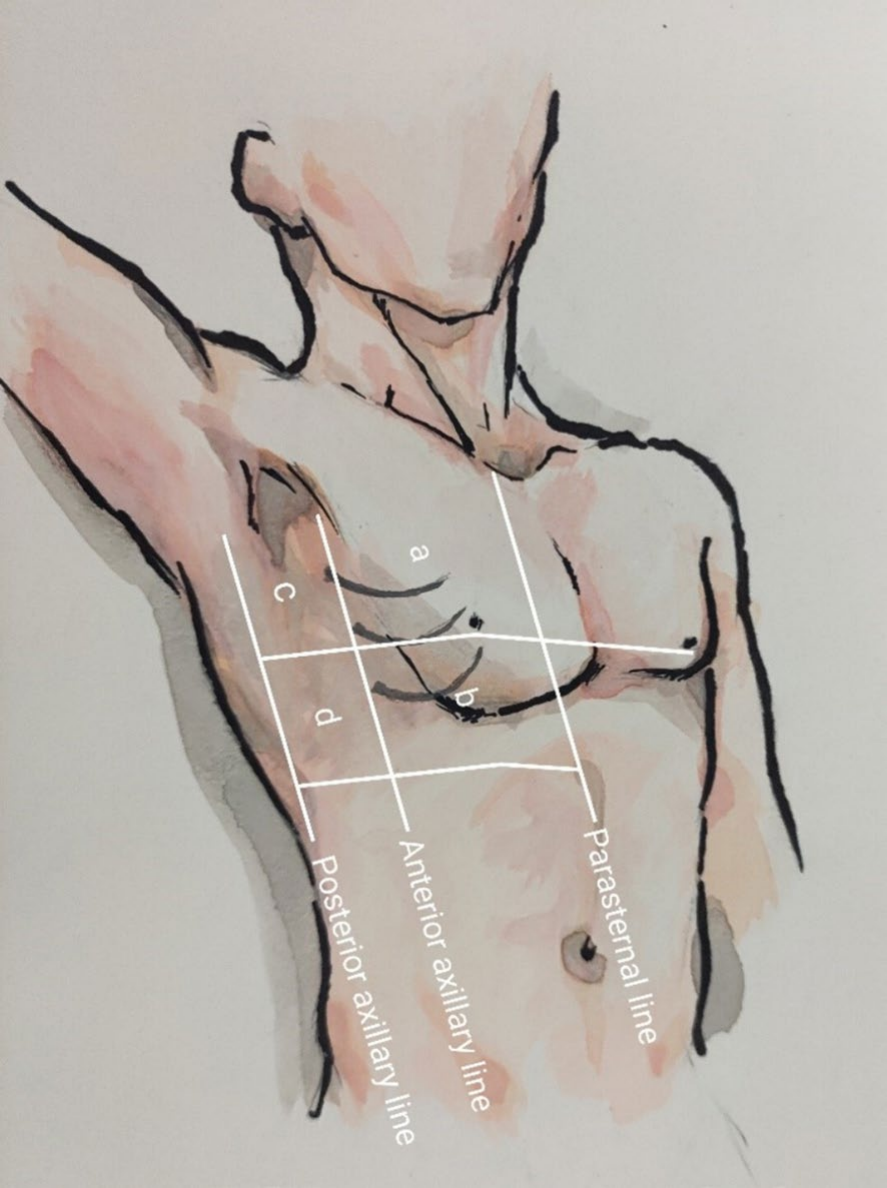
Zones followed for lung ultrasound examination

**Fig. 3:**
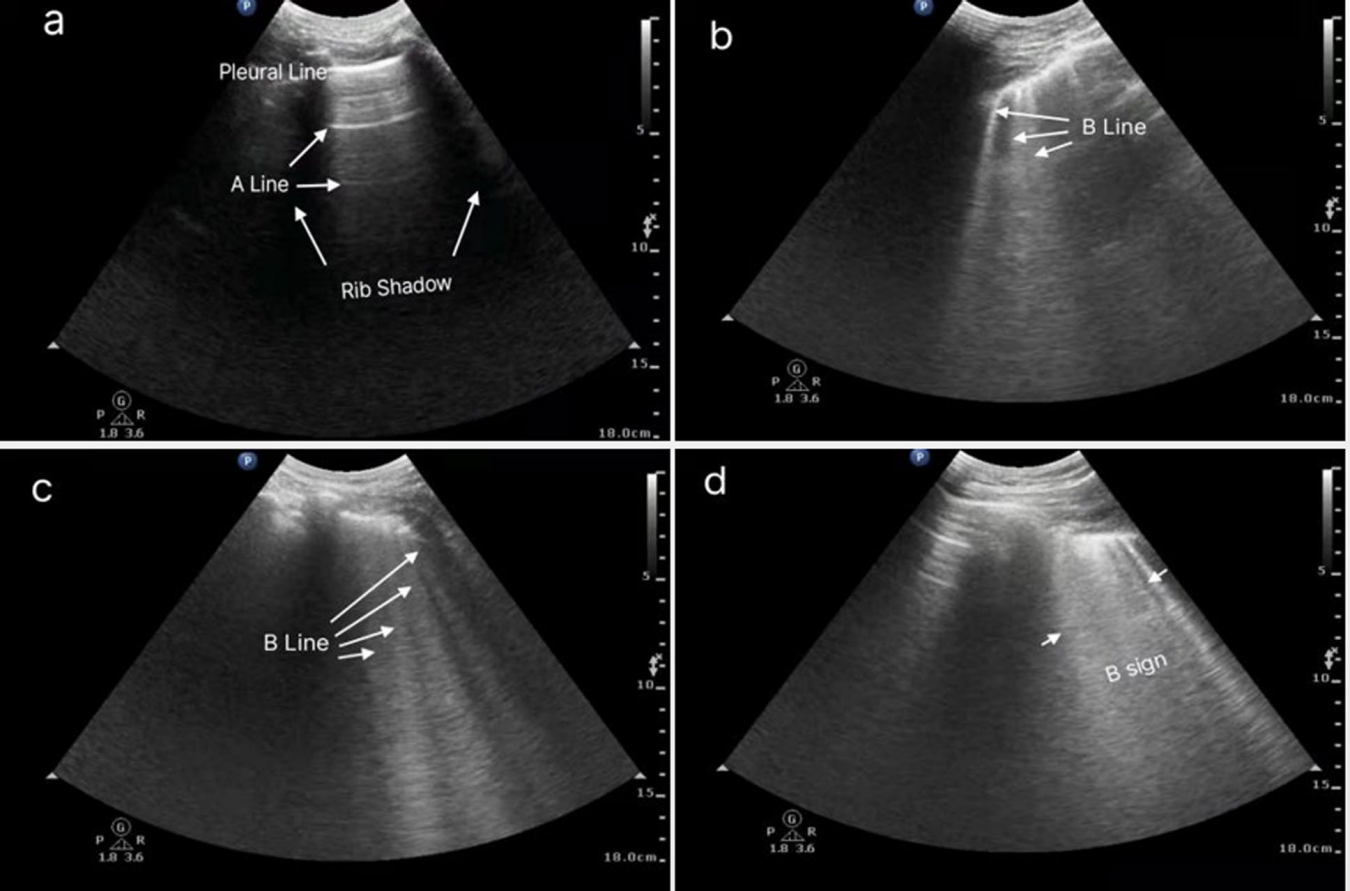
Example of lung ultrasound B-line score. **a**, **b**, **c**, and **d** correspond to scores of 0, 3, 4, and 5, respectively

The B-line is described as a comet-tailed artifact originating from the pleural line, which extends vertically from the pleura to the bottom of the screen without attenuation, moving with sliding of the lung. Multiple B-line fusions present as diffuse subpleural hyperechoic shadows [16]. A portable ultrasound convex array probe (probe Model C5-1, Phillips-CX50, Andover, MA, USA) was used for lung ultrasound, with the patient placed in the supine position. Lung ultrasound was performed by the same sonographer for every case using the classic 8-zone method. In each area, the probe was first placed perpendicular to the ribs, and the B-line count was conducted once the bat sign was identified. The probe was then placed parallel to the intercostal space, and the maximum number of B-lines was counted twice. In particular, the probe was adjusted three times in each region to obtain the maximum value (Fig. 2). B-line scoring rules are as follows: A line or no B-line, 0 points; each B-line, 1 point; fusion B-line accounting for 50% of the screen area, 5 points; an area of 75%, 8 points; full screen B-line, 10 points; total score, 0–80 points (Fig. 3).

**Fig. 4:**
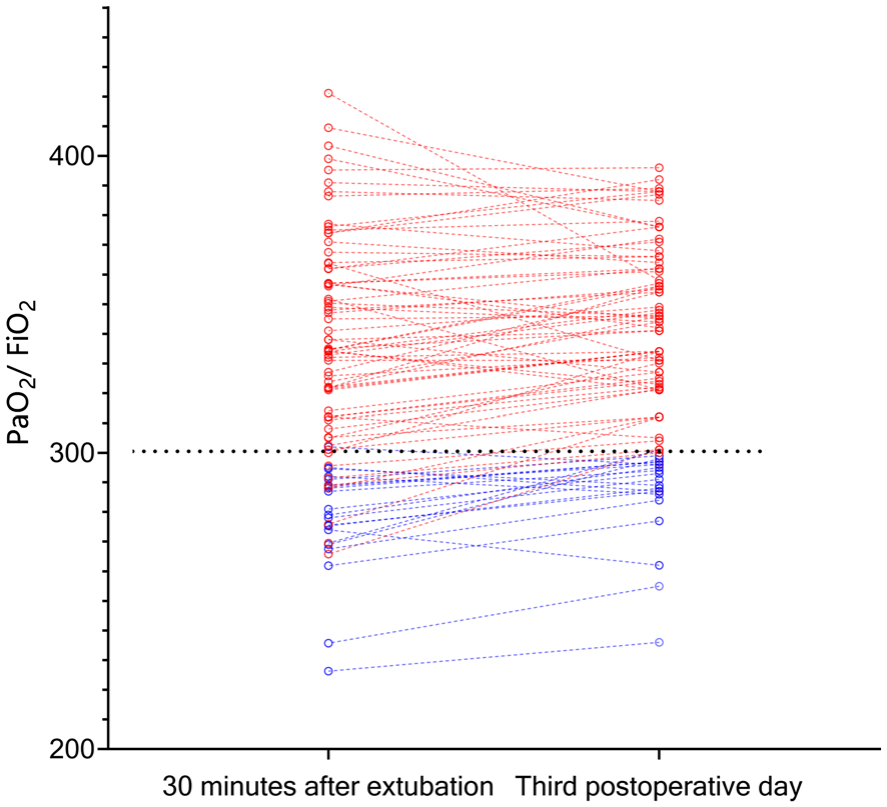
Change in PaO_2_/FiO_2_ from 30 min after extubation to postoperative day 3. The blue and red lines indicate PaO_2_/FiO_2_ < 300 and ≥ 300 on the third postoperative day, respectively

### Data collection

The B-line score was recorded 30 min after extubation, and the PaO_2_/FiO_2_ values were recorded 30 min after extubation and on the 3rd day after surgery. Data regarding patient demographics (age, sex, body mass index, ASA classification, New York Heart Association [NYHA] classification at admission, liver and kidney function indices, hemoglobin level, history of smoking) and surgical factors (operative method, procedure duration, infusion volume) were collected, as well as information about each patient’s postoperative hospital stay.

### Statistical analysis

Based on a previously reported incidence of PPI in thoracic surgery of 20% [22] and the anticipated sensitivity and specificity of lung ultrasound of 95% with a 10% margin of error, the estimated sample size was 91. Considering 5% attrition during the study, a total of 95 patients were enrolled. Data processing was performed using IBM SPSS Statistics 20 (IBM Corporation, Armonk, NY, USA) and GraphPad Prism Software version 8.0.2 (GraphPad Software, La Jolla, CA, USA). The data were checked for normality using the Shapiro Wilks test, and the continuous variables were expressed as the mean and standard deviation or the median and interquartile range accordingly. Student’s *t* test and Mann‒Whitney *U* rank-sum tests were used for comparison of normal and nonnormally distributed data, respectively. Categorical variables were expressed as numbers and percentages, and data differences between the two groups were compared using a chi-square test. The difference in PaO_2_/FiO_2_ at 30 min after extubation and on postoperative Day 3 was compared using a paired *t* test. Linear regression models were used to assess the interdependence of baseline parameters, B-line scores and PaO_2_/FiO_2_ on postoperative Day 3. Variance inflation factors (VIFs) were calculated to assess continuous variable covariance. A stepwise forward logistic regression model (*p* for entry < 0.1, for exit > 0.1) was constructed to identify independent risk factors for postoperative pulmonary insufficiency. Odds ratio values were calculated, and the best predictive values for continuous variables were calculated based on recipient operating characteristic curves and the Youden index. The model fit was assessed using the Hosmer and Lemeshow statistic. A *P*-value < 0.05 was considered statistically significant (see Additional file 1).

## Results

Ninety-five patients were initially enrolled in the study; six of these patients were excluded from the final analysis because of loss to follow-up (*n* = 3), poor visualization conditions (*n* = 2), and the presence of postoperative atrial fibrillation (*n* = 1). Consequently, the final study population included 89 patients, of whom 26 (29.2%) had PaO_2_/FiO_2_ < 300 within 30 min after extubation and 63 had PaO_2_/FiO_2_ ≥ 300. Twenty (22.47%) patients had PaO_2_/FiO_2_ < 300 on postoperative Day 3 (PPI group); the remaining 69 patients had PaO_2_/FiO_2_ ≥ 300 (normal group). Notably, although overall, the patients improved in terms of PaO_2_/FiO_2_ on the third postoperative day compared to 30 min after extubation (325.82 ± 40.85 vs. 331.84 ± 34.87; *p* < 0.001), there were still 19 subjects with persistent pulmonary insufficiency in both periods, 7 with improved lung function on the third postoperative day and 1 with decreased lung function. PaO_2_/FiO_2_ < 300 at 30 min after extubation lasted until postoperative Day 3 in 19 of 26 (73.08%), while only 1 of 63 patients (1.58%) with pre-existing PaO_2_/FiO_2_ ≥ 300 had worsening pulmonary function resulting in PPI (Table 2, Fig. 4).

**Fig. 5:**
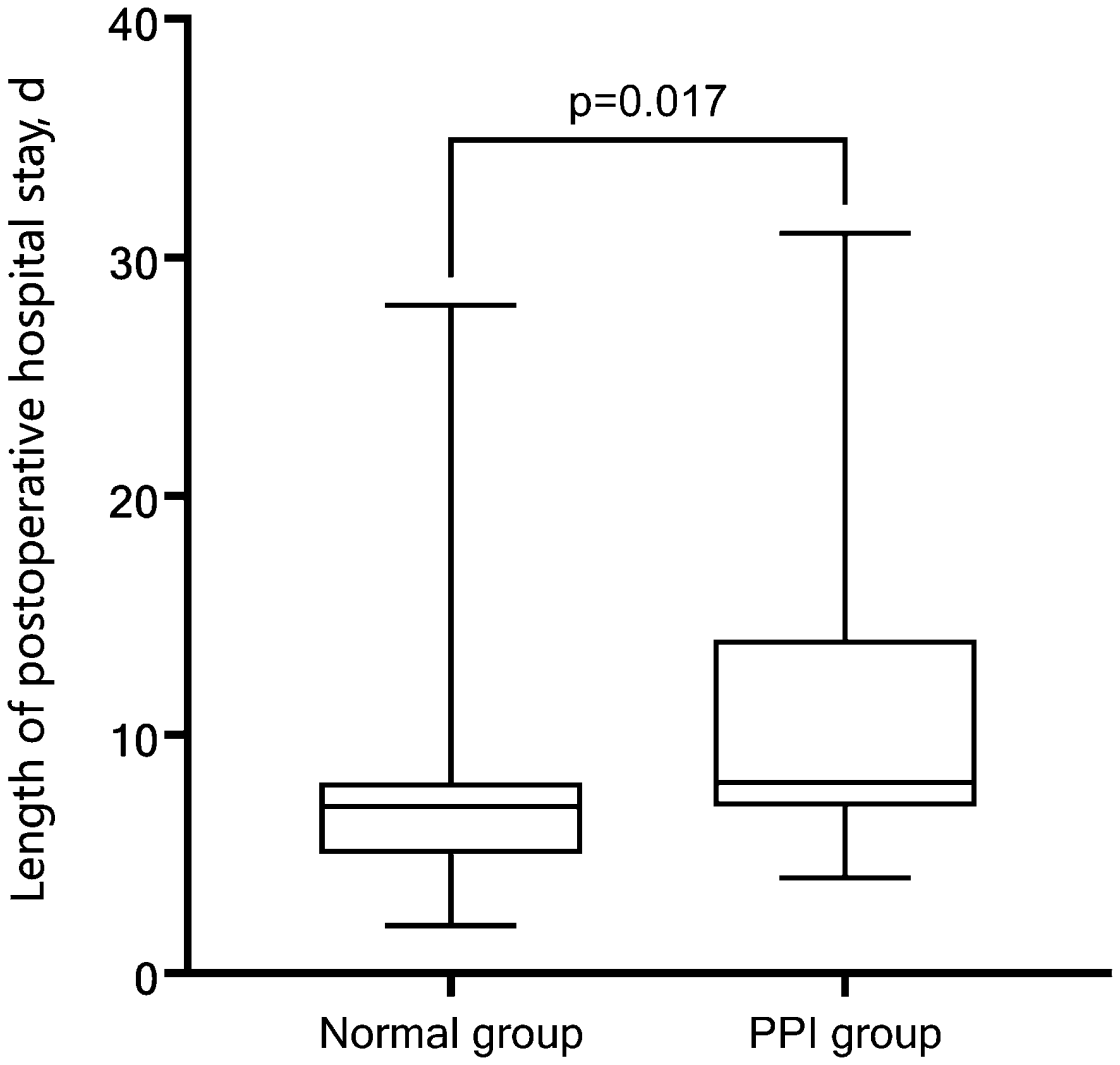
Length of postoperative hospital stay in the normal group (PaO_2_/FiO_2_ ≥ 300) and PPI group (PaO_2_/FiO_2_ < 300)

In the baseline data, there were significant differences between the two groups in terms of age and albumin levels. Patients in the PPI group were older and had lower albumin levels and a higher proportion of ASA class 3 and HYHA class 3 (Table 1). Regarding the perioperative characteristics, the PPI group had longer operative times, greater infusion volumes and longer postoperative hospital stays than the normal group. [7 (5–8) vs. 8 (7–15) *p* = 0.017] (Table 2) (Fig. 5).

**Table 1:** Baseline characteristics of the patients by group

Parameters	All patients (*N* = 89)	PaO_2_/FiO_2_ ≥ 300 (NORM = 69)	PaO_2_/FiO_2_ < 300 (PPI = 20)	*p*-value^*^
Age (years)	58 (49.5–65.5)	56 (47–67)	71 (63–74)	**0.001**
Male, *N* (%)	42 (47.2)	33 (47.82)	9 (45)	0.803
Smoker, *N* (%)	22 (24.72)	14 (20.29)	8 (40)	0.132
NYHA 3, *N* (%)	15 (16.85)	4 (5.8)	11 (55)	** < 0.001**
ASA 3, *N* (%)	18 (20.22)	10 (14.49)	8 (40)	**0.029**
Hemoglobin level (g/L)	130.45 ± 15.76	132 ± 15	125 ± 16	0.069
Albumin level (g/L)	41.25 ± 4.67	42.10 ± 3.85	38.31 ± 6.03	**0.001**
Serum creatinine level (umol/L)	64 (54–74.5)	62 (54–75)	67.4 (54–71.5)	0.898
Urea nitrogen level (mmol/L)	5.05 ± 1.45	5.04 ± 1.5	5.11 ± 1.28	0.842
Body mass index (kg/m^2)^	22.73 ± 2.71	22.92 ± 2.73	22.06 ± 2.71	0.21

Data are expressed as median (interquartile range), *N* (%), or mean ± standard deviation, as appropriate

PPI: postoperative pulmonary insufficiency; PaO_2_, partial pressure of arterial oxygen; FiO_2_, fraction of inspired oxygen; NYHA, New York Heart Association; ASA, American Society of Anesthesiology; NYHA 3: NYHA 3 class at administration

*p-value indicates the comparison between the NORM and PPI groups

Bold values are statistically significant

**Table 2:** Perioperative characteristics of the patients by group

Parameters	All patients (*N* = 89)	PaO_2_/FiO_2_ ≥ 300 (NORM = 69)	PaO_2_/FiO_2_ < 300 (PPI = 20)	*p*-value^*^
Lobectomy, *N* (%)	35 (39.33)	25 (36.23)	10 (50)	0.267
Procedure duration (min)	90 (70–155)	80 (65–125)	175 (100–210)	** < 0.001**
Infusion volume (mL)	800 (690–1000)	750 (650–850)	1000 (1000–1575)	**0.001**
B-line score	9 (5–13)	7 (5–10)	16 (13–21)	** < 0.001**
PaO_2_/FiO_2_ 30 min after extubation	325.82 ± 40.85	339.81 ± 34.27	277.53 ± 19.01	** < 0.001**
PaO_2_/FiO_2_ on postoperative day 3	331.84 ± 34.87	345.31 ± 26.06	286.35 ± 16.38	** < 0.001**
Length of postoperative stay (day)	7 (5–10)	7 (5–8)	8 (7–15)	**0.017**

*p-value indicates the comparison between the NORM and PPI groups

Bold values are statistically significant

**Fig. 6:**
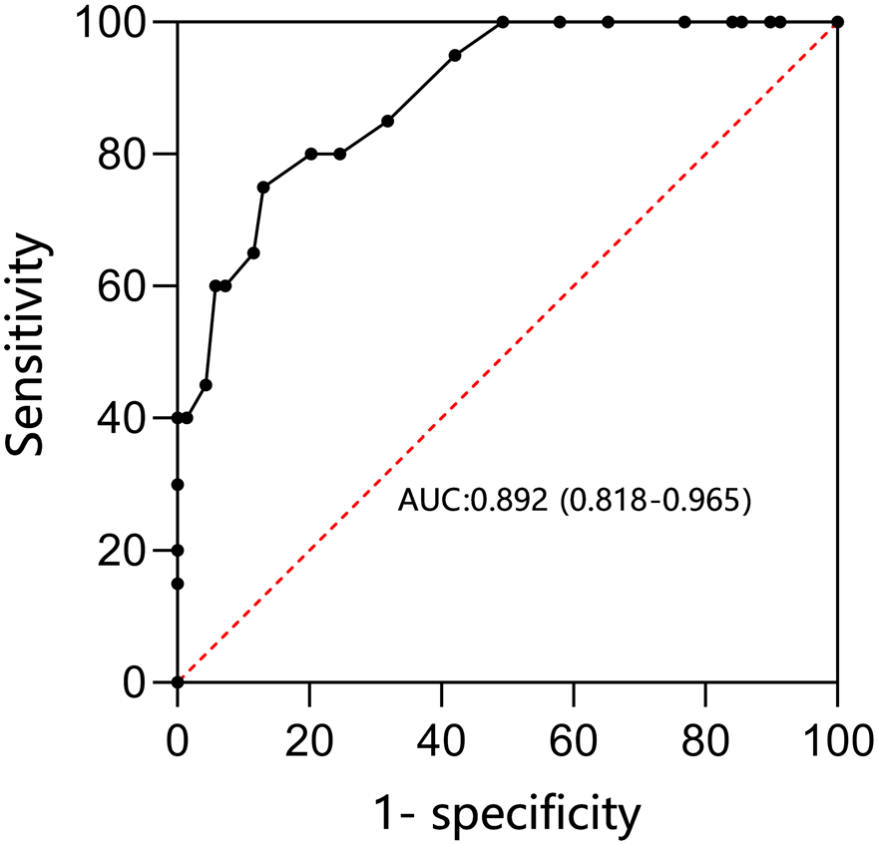
Area under the receiver operating characteristic curve for the ability of B-line score to predict PaO_2_/FiO_2_ < 300 on postoperative day 3

The B-line score was significantly higher in the PPI group than in the normal group (16; IQR 13–21 vs. 7; IQR 5–10; *p* < 0.001), corresponding to PaO_2_/FiO_2_ on postoperative Day 3 (286.35 ± 16.38 vs. 345.31 ± 26.06; *p* < 0.001), respectively. The area under the receiver operating characteristic curve for the correlation between the B-line score and PaO_2_/FiO_2_ < 300 was 0.892 (95% CI 0.818 to 0.965; *p* < 0.001) (Fig. 6). The optimal cutoff value for the B-line score as a predictor of PaO_2_/FiO_2_ < 300 was 12 (sensitivity of 77.5%; specificity of 66.7%).

**Fig. 7:**
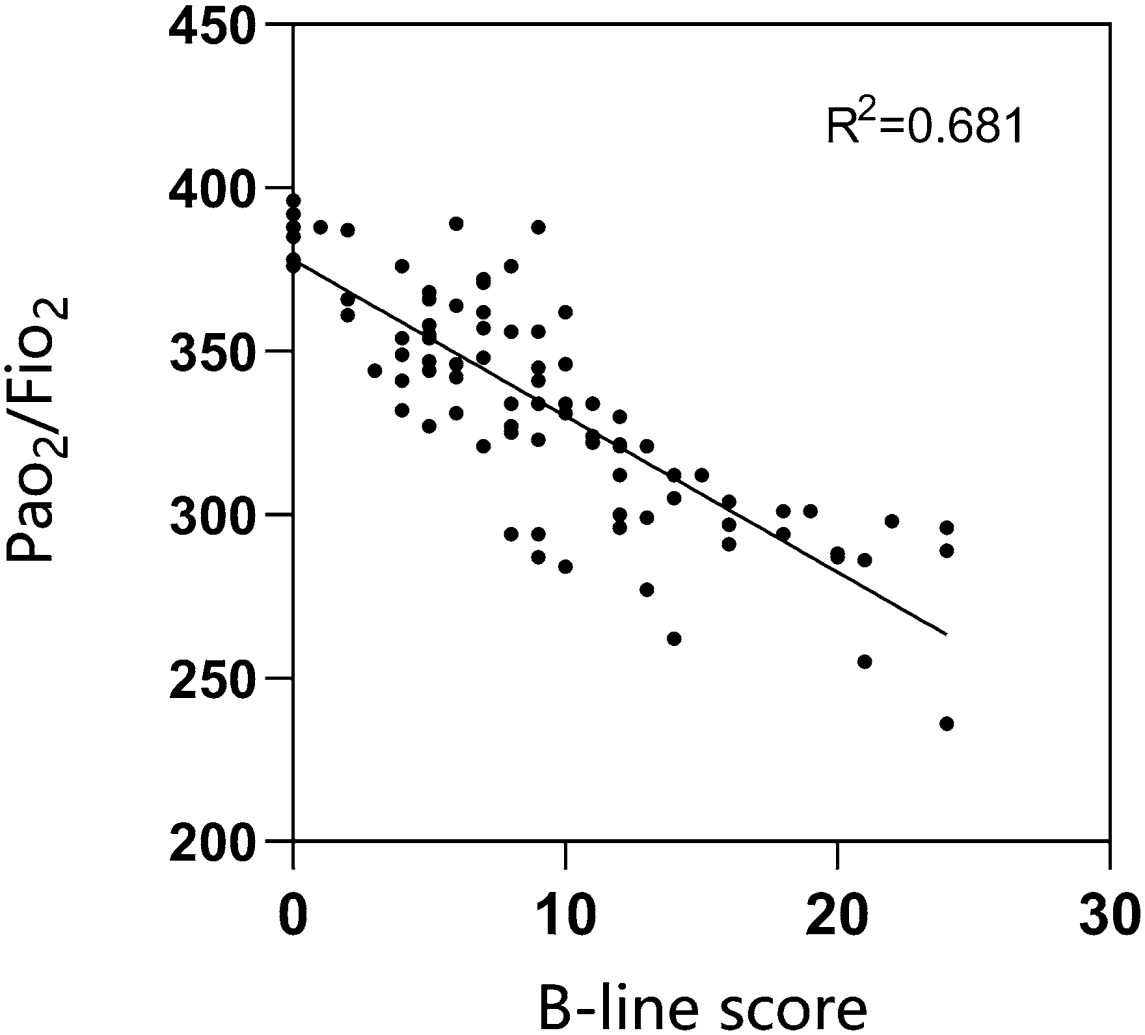
Correlation between B-line score and PaO_2_/FiO_2_ on postoperative day 3

The VIFs of all continuous variables were less than 5, and there was no significant collinearity. In the forward stepwise model building, the B-line score and NYHA 3 class were retained (Table 3). This suggests that NYHA class 3 and the B-line score at 30 min after extubation are independent risk factors for the development of PPI, corresponding to odds ratios of 8.572 and 1.349, respectively (*p* = 0.007 and *p* < 0.001). The Hosmer Lemeshow assessment of fit indicated good calibration. Linear regression models showed that a variety of parameters, including the B-line score, were significantly associated with the oxygenation index on the third postoperative day, with higher B-line scores and a longer procedure duration leading to lower PaO_2_/FiO_2_ and higher postoperative PaO_2_/FiO_2_ with moderate rehydration in patients in NYHA class 1–2 class at administration (Table 4, Fig. 7).

**Table 3:** Odds ratios of predictors for PPI retained in the multivariate analysis

Variable	Odds ratio	*p*-value	95% CI
B-line score	1.349	< 0.001	1.154–1.578
NYHA 3	8.572	0.007	1.738–42.276

NYHA, New York Heart Association; CI: confidence interval

**Table 4:** Liner Regression Analysis of Variables Potentially Associated With PaO_2_/FiO_2_

Variable	Coefficient	*p*-value	95% CI	Importance
B-line score	− 5.020	< 0.001	− 6.003 to 4.036	0.76
Procedure duration	− 0.179	< 0.001	− 0.265 to 0.092	0.123
Infusion volumes	0.022	0.013	0.005 to 0.039	0.047

## Discussion

The main objective of this study was to estimate the value of lung ultrasonographic variables in the early postoperative stage for predicting PPI. According to the results of Touw’s studies, lung ultrasound was valuable in screening for postoperative pulmonary pathologies after cardiac surgery with superiority compared to chest X-rays [23]. A recent study reported that among non-ICU postoperative patients, the B-line score can be a predictor of not only respiratory failure but also other PPIs. Similarly, French studies also showed that patients postoperatively admitted to the ICU more frequently needed postoperative ventilatory support and had a lower PaO_2_/FiO_2_ ratio if their B-line score was at least 10 immediately after admission [24]. All the above studies demonstrated that the quantitative B-line score based on lung ultrasound is a valuable tool with high sensitivity and good specificity in the identification of developing complications or patients at risk. However, there is no study on the application of early postoperative B-line scores to the prediction of pulmonary insufficiency in patients undergoing thoracic surgery. In this study, patients with pulmonary insufficiency (PaO_2_/FiO_2_ < 300) differed significantly from those in the normal group (PaO_2_/FiO_2_ ≥ 300) in terms of B-line score, age, proportion of ASA3 and NYHA 3, procedure duration, infusion volumes, and albumin level. Patients with PPI also had a significantly longer postoperative hospital stay than those without pulmonary insufficiency. Further analysis showed a significant correlation between postoperative PaO_2_/FiO_2_ and B-line score, procedure duration, and infusion volumes. These results revealed that the B-line score is a reliable index for predicting the occurrence of postoperative pulmonary insufficiency and that procedure duration and infusion volumes are important factors affecting postoperative oxygenation.

In this study, NYHA3 patients were found to be significantly overrepresented in the pulmonary insufficiency group, similar to previous studies assessing pulmonary congestion in patients with heart failure [25]. The myocardium is in an inhibited state during anesthesia, and the increased pulmonary artery pressure promotes an increase in extravascular lung fluid; this leads to cell edema and metabolic disorders and can lead to respiratory failure in severe cases. Fluid balance is an important factor to consider in this setting, as sympathetic nerve inhibition during anesthesia weakens the kidney’s ability to regulate body fluid. In cases of fluid overload, the balance between tissue fluid generation and reflux in the lung is upset, resulting in pulmonary edema. In one study of critically ill patients, fluid resuscitation was guided through the assessment of fluid responsiveness using B-line quantitative counting. Previous studies have found that intraoperative fluid infusions of more than 5000 ml are a risk factor for postoperative pulmonary complications (PPCs) [1]. There were no significant results in this trial to support this finding, even though appropriate fluid rehydration in the study population improved the oxygenation indices, which were not in conflict. The reason for this is, on one hand, that very few patients require large amounts of rehydration within the constraints of the type of surgery, which leads to a lack of samples in this category; on the other hand, in patients with (absolute or relative) blood volume deficiencies, appropriate fluid replacement improves the perfusion of vital tissues and organs and helps to establish a ventilation–to–blood-flow ratio suitable for gas exchange. The amount of fluid administered as a correlate of PPI is highly variable on an individual basis. The compensatory capacity of fluid-regulating organs such as the heart, lungs and kidneys needs to be taken into account in different patients. In contrast, the B-line score can be implemented in all types of patients as long as the observed conditions are met. For the purposes of this study, the B-line score is a more valuable predictor of PPI than the rehydration volume.

Further analysis of patients with PPI showed that among the 20 patients with pulmonary insufficiency at postoperative Day 3, the pulmonary insufficiency had already been demonstrated 30 min after extubation. Correspondingly, the B-line scores of these patients were increased. This suggests that the oxygenation index at 30 min after extubation is also predictive of lung function status on postoperative Day 3. Nevertheless, compared to the B-line score, the index still has the following limitations: first, the presence of residual drugs (opioids, inotropes, central sedatives) in the early postoperative period, which inhibit the patient's breathing; and second, the limitation of the patient's respiratory movements due to acute postoperative pain. All these factors cause failure of the oxygenation index during this period to accurately reflect the patient’s real-time lung function status. In contrast, visual monitoring of the patient's postoperative pulmonary (alveolar and interstitial) edema and exudation by B-line scoring allows a quantitative assessment of the patient's pulmonary ventilation status as a whole. Although most of the 19 patients had an increase in PaO_2_/FiO_2_ 3 days after surgery, the ratio was still < 300. This suggests that for patients with decreased PaO_2_/FiO_2_ in the early postoperative period, it is difficult for recovery to occur through self-compensation regulation, and the postoperative hospital stay will be prolonged accordingly. Unlike previous studies in which many factors influenced postoperative lung function [26], the B-line score, an independent risk factor, was the most relevant in this study. Combined with the significant differences in patient characteristics at baseline and perioperative characteristics (age, NYHA 3, ASA 3, albumin level, procedure duration, infusion volumes), the B-line score is likely to be a combination of these factors, making it a more feasible predictor of change in perioperative parameters than in other studies.

There are several limitations to this study. First, the fact that pulmonary insufficiency is defined differently from PPCs leads to limitations in the application of the study results, but early detection of pulmonary insufficiency is still clinically relevant for improving prognosis. Second, the renal function among the study patients was generally good, so it is unclear whether the B-line score was predictive of postoperative lung function in patients with poor renal function. Finally, there are few objective indicators (hemodynamic parameters and myocardial enzyme spectrum indicators) of cardiac function, and therefore, the potential effect of this factor on postoperative lung function needs further investigation.

## Conclusion

In summary, the B-line score assessed by lung ultrasound is a simple, safe, repeatable test that can be used to predict early postoperative pulmonary function in patients undergoing thoracic surgery.

## Supplementary Information

**Additional file 1**. STROBE Statement—checklist of items that should be included in reports of observational studies.

## Data Availability

The datasets used and/or analysed during the current study are available from the corresponding author on reasonable request.

## Abbreviations

PPI: Postoperative pulmonary insufficiency
ROC: Receiver operating characteristic
VIF: Variance inflation factors
PPCs: Postoperative pulmonary complications
